# A Novel Malaria Vaccine Candidate Antigen Expressed in *Tetrahymena thermophila*

**DOI:** 10.1371/journal.pone.0087198

**Published:** 2014-01-29

**Authors:** Graeme J. M. Cowan, Ulrike Bockau, Janna Eleni-Muus, Ingo Aldag, Kay Samuel, Alison M. Creasey, Marcus W. W. Hartmann, David R. Cavanagh

**Affiliations:** 1 Institute of Immunology and Infection Research, Centre for Immunity, Infection and Evolution, University of Edinburgh, Edinburgh, United Kingdom; 2 Cilian AG, Münster, Germany; 3 Cell Therapy Group, Scottish National Blood Transfusion Service, Edinburgh, United Kingdom; Johns Hopkins Bloomberg School of Public Health, United States of America

## Abstract

Development of effective malaria vaccines is hampered by the problem of producing correctly folded *Plasmodium* proteins for use as vaccine components. We have investigated the use of a novel ciliate expression system, *Tetrahymena thermophila*, as a *P. falciparum* vaccine antigen platform. A synthetic vaccine antigen composed of N-terminal and C-terminal regions of merozoite surface protein-1 (MSP-1) was expressed in *Tetrahymena thermophila*. The recombinant antigen was secreted into the culture medium and purified by monoclonal antibody (mAb) affinity chromatography. The vaccine was immunogenic in MF1 mice, eliciting high antibody titers against both N- and C-terminal components. Sera from immunized animals reacted strongly with *P. falciparum* parasites from three antigenically different strains by immunofluorescence assays, confirming that the antibodies produced are able to recognize parasite antigens in their native form. Epitope mapping of serum reactivity with a peptide library derived from all three MSP-1 Block 2 serotypes confirmed that the MSP-1 Block 2 hybrid component of the vaccine had effectively targeted all three serotypes of this polymorphic region of MSP-1. This study has successfully demonstrated the use of *Tetrahymena thermophila* as a recombinant protein expression platform for the production of malaria vaccine antigens.

## Introduction

Malaria continues to be a major public health challenge, particularly among children and pregnant women in sub-Saharan Africa [1]. Prompt medical intervention is unavailable in many malaria-endemic regions due to limitations in healthcare infrastructure, organization and resources. In light of the continuing emergence of drug-resistant strains of *Plasmodium falciparum*, there is a pressing need for effective vaccines against malaria [2].

There are a limited number of candidate malaria vaccine antigens in various stages of development, from early proof-of-concept studies to late-phase clinical trials [3], [4]. An extensive array of expression platforms have been used to generate vaccine antigens against malaria, including synthetic peptides, viral delivery systems, bacteria, transgenic plants or animals, insect cells, mammalian cell lines and yeast [5]–[9]. The production of *Plasmodium* proteins for use in subunit vaccines using heterologous expression systems presents a number of challenges, since efficient expression of correctly folded proteins may be precluded by the inherent characteristics of many *P. falciparum* genes and their products, such as repetitive sequence content, large open reading frames, complex disulfide bonding patterns and the high AT content of *P. falciparum* DNA [10], [11]. In this study we investigated the use of a promising protozoan protein expression system similar to *Plasmodium falciparum* parasite’s own biosynthetic machinery.

*Tetrahymena thermophila* is a ciliated protozoan belonging to the eukaryotic clade alveolata that also includes *Plasmodium spp*. [12]. It shares some inherent characteristics with *P. falciparum,* such as similar codon usage bias and the production of a number of large and structurally complex proteins [11], [12]. As a biotechnological expression system, *T. thermophila* cells grow rapidly to high densities in simple, inexpensive media. The fermentation process uses conventional bioreactor equipment typically used for *E. coli* or yeast systems, and it is readily up-scalable to large volumes suitable for bioprocess production [13], [14]. Finally, as there is no evidence that *T. thermophila* harbours any pathogenic viruses or pyrogens [15], proteins expressed in this system should be biologically safe and free from human pathogens.

It has previously been demonstrated that *T. thermophila* can express genes from heterologous species. Initially, a surface antigen of the fish ectoparasite *Ichthyophthirius multifiliis* was expressed on the surface of the ciliate [16]. Subsequently, the full-length expression of the GPI-anchored circumsporozoite protein of *Plasmodium falciparum* and surface expression of the C-terminal 19 kDa region of merozoite surface protein 1 (MSP-1) have been achieved [17], [18]. More recently, human enzymes have been expressed and purified from *T. thermophila* [19]. However, to date successful production and immunogenicity testing of a soluble *Plasmodium* protein expressed in *Tetrahymena* has not been described.

MSP-1 is the most abundant surface protein of the invasive merozoite stage of the *P. falciparum* life cycle, making up 40% of the GPI-anchored surface protein coat [20], [21]. The molecule can be divided into 17 blocks based on sequence diversity from primary sequence alignments from different strains [22]. The N-terminal Block 2 region of MSP-1 is by far the most polymorphic region of the molecule, with hundreds of known variant sequences from globally diverse parasite isolates [23], [24]. MSP-1 Block 2 represents a promising target for malaria vaccine development, since several seroepidemiological studies have shown that individuals with antibodies to MSP-1 Block 2 have reduced risk of subsequent clinical malaria episodes [25]–[29]. Antibodies to MSP-1 Block 2 have also been shown to inhibit parasite growth in antibody-dependent cellular inhibition (ADCI) assays [30]. The allelic diversity of MSP-1 Block 2 gives rise to the possibility that variant-specific immune responses may be elicited that would fail to provide protection against all parasite genotypes of a given population. To overcome this problem, a synthetic hybrid antigen that covers all known sequence diversity of the MSP-1 Block 2 region was designed and has been used in this work [31]. This MSP-1 hybrid antigen also incorporates the conserved Block 1 region of MSP-1 which improves the immunogenicity of the construct by inclusion of cognate T-cell help, since this region has been shown to contain human and mouse T-cell epitopes [32], [33].

MSP-1_19_ is the C-terminal part of MSP-1, also known as MSP-1 Block 17 and is a highly conserved protein domain, rich in cysteine residues and comprised of two EGF-like motifs [34]–[36]. Antibodies that bind to certain epitopes of MSP-1_19_ can inhibit invasion of red blood cells by the merozoite stage of the parasite, through inhibition of the proteolytic processing of MSP-1 [37]–[39]. Antibodies to MSP-1_19_ purified from hyperimmune human serum can inhibit parasite invasion *in vitro* [40]. In several seroepidemiological studies, naturally exposed individuals with antibodies to MSP-1_19_ have shown reduced incidence of clinical malaria [2], [27], [41], [42].

Previous human clinical studies using MSP-1_19_ derived vaccine antigens have shown low immunogenicity in humans [43] and most subsequent trials have utilized a larger C-terminal fragment, MSP-1_42_ that exhibits greater immunogenicity [44]. The region of MSP-1 used in this study included MSP1_19_, plus 35 C-terminal residues of Block 16 present immediately upstream of MSP-1_19_, which contains human T-cell epitopes and has been shown to be responsible for strong dimerization of *P. vivax* C-terminal MSP-1 constructs [45]. We hypothesized that inclusion of this upstream Block 16 sequence would improve the immunogenicity of the construct, as an MSP-1_19_ vaccine construct expressed in baculovirus containing the majority of this Block 16 sequence, plus the N-terminal Block 1 region, showed superior pre-clinical immunogenicity compared to MSP-1_19_ alone [46].

In this study we have investigated the use of *Tetrahymena thermophila* as an expression system for *Plasmodium* antigens by expressing and purifying a synthetic vaccine antigen, MSP-1-BBM, comprised of the conserved MSP-1 Block 1 region, the multi-allelic MSP-1 Block 2 hybrid sequence, the dimerization region of Block 16, and Block 17 of MSP-1. The aim is to develop an MSP-1 based vaccine targeting both conserved and polymorphic regions of this major merozoite antigen, which can elicit antibodies that are strain-transcending and functionally effective against the malaria parasite. We present the results of immunogenicity testing in mice of this purified soluble vaccine antigen from *T. thermophila*.

## Materials and Methods

### Ethics Statement

All *in vivo* experiments were carried out in accordance with the Animals (Scientific Procedures) Act 1986 and conformed to the Recommendations from the Declaration of Helsinki and the Guiding Principles in the Care and Use of Animals. The University of Edinburgh Ethical Review Committee approved the project license on 3rd July 2006, reference number PL 13-06, under which all mouse experimentation was performed. Mice were humanely killed by Schedule 1 methods, in accordance with licensing requirements. Group sizes were assessed prior to experimentation to limit animal numbers while maintaining sufficient statistical power.

### Design and Cloning of MSP-1-BBM

The synthetic MSP-1-BBM antigen was designed as a four-component construct encoding the MSP-1 hybrid sequence composed of MSP-1 Block 1 and synthetic sequences covering all three MSP-1 Block 2 serotypes [31], fused to residues 1572–1702 of 3D7 strain MSP-1 (PlasmoDB.org ref. PFI1475W). The signal sequence from MSP-1 was added to promote secretion of the antigen [18]. The resulting sequence was codon optimized for expression in *Tetrahymena thermophila* and synthesized by GeneArt AG, Regensburg, Germany. All potential N-glycosylation sites were removed by converting appropriate asparagine residues to glutamine. To facilitate cloning of the open reading frame encoding the antigen, an EcoRV restriction site was inserted directly downstream of the start methionine within the MSP-1 signal sequence and a BglII site was inserted downstream of the stop codon.

The MSP-1-BBM coding sequence was cloned by restriction/ligation at the BglII and EcoRV sites into the expression cassette of the pDL325 donor plasmid [18] and amplified in *E. coli* DH10B cells (Invitrogen GmbH, Karlsruhe, Germany) under kanamycin selection. The final expression plasmid p22X-MSP-1-BBM was generated by the transfer of the expression cassette into the integrative acceptor plasmid p22X by Cre/Lox recombination [18], [47].

The p22X plasmid is similar to pKOIX [18], but the DHFR-TS integration sites were replaced by two 1.5 kb regions of a protease gene locus (NCBI Genbank Accession XM_001027342). The resulting vector is ∼7.5 kb in size and contains a *loxP* site for Cre-mediated integration of the expression cassette downstream of the *neo2* selection cassette [18], [48]. Further details of the constructs are available from the authors.

### Ciliate Strains, Cultivation and Transformation

*T. thermophila* inbred strains B1868/4 and B2068/1 were used as transformation hosts. Conjugating cells were transformed via biolistic bombardment using standard protocols [49], [50]. Afterwards, individual transformants were further cultivated at 30°C without agitation, in 1.5 mL SPP-medium supplemented with increasing concentrations of the antibiotic paromomycin (from 100 µg mL^−1^ to 1000 µg mL^−1^) for several weeks to support the allelic assortment process. Small-scale cultivation was performed in 1.5–10 mL SPP medium at 30°C and 80 rpm in a Multitron AJ incubation shaker (Infors AG, Bottmingen, Switzerland).

### SDS-PAGE and Western Blots

Whole-cell lysates and cell-free culture supernatant of recombinant *T. thermophila* induced with cadmium chloride (5 µg mL^−1^ for 6–24 hours) were screened for MSP-1-BBM expression by SDS-PAGE and Western blotting using standard methods [51]. Monoclonal antibodies 12.2 and 12.8, which recognize MSP-1 Block 2 and MSP-1_19_ respectively, were kindly provided by Dr. Jana McBride and used as primary antibodies [52]. Equal volumes of serum from all mice immunized with MSP-1-BBM were mixed to produce a pool. MSP-1 Block 2 hybrid protein [31] and MSP1_19_-GST fusion protein [53] were resolved by SDS-PAGE and blotted onto nitrocellulose membrane, then probed with a 1∶1000 dilution of pooled immune serum followed by anti-mouse IR-Dye680 conjugated secondary antibody (Licor Biosciences Ltd., Cambridge, UK). All Western blots were visualized using a Licor Odyssey scanner.

### Isocitrate Dehydrogenase (ICDH) Assay

Isocitrate dehydrogenase was selected as marker for cell damage in *T. thermophila* cultures. The assay was performed spectrophotometrically according to the manufacturer’s recommended protocol (Biochemika, Boehringer Mannheim GmbH, Germany).

### Fermentation of Recombinant *T. thermophila* and Cell Harvesting

Batch fermentation of *T. thermophila* was conducted in a Biostat UD 50 fermentor (Sartorius, Goettingen, Germany) at 50-liter scale. The fermentor was inoculated with 24×10^3^ cells mL^−1^ and cells were grown in SPP-based medium supplemented with 2.5 µg mL^−1^ E-64 (Peptanova GmbH, Sandhausen, Germany). The temperature was maintained at 30°C and pO_2_ was controlled at 20% of the air saturation level by stirrer speed (150–250 rpm) and air flow (10–35 L min^−1^). The pH value was not regulated, with an initial pH of 7.0.

MSP-1-BBM expression was induced by adding 5 µg mL^−1^ cadmium chloride to the culture after 17 h of growth (cell density of 0.25×106 cells mL^−1^). 40 h after inoculation (cell density: 1.7×10^6^ cells mL^−1^), the culture broth was harvested cell-free by filtration with a hollow fiber module (PES, 1 mm lumen, 3200 cm^2^ area, 0.5 µm cut off; Spectrum Laboratories Inc., USA), producing 47 L of supernatant from 50 L culture. The filtrate was concentrated to 2 liters using Sartocon Slice cassettes (Sartorius, 10 kDa cut off) and concentrate was washed with 10 mM Phosphate buffer (pH 7.0) at the end of the concentration. Finally, 536.4 g ammonium sulfate were added to 2 liters of concentrate and incubated for 20 minutes on ice. Precipitated protein was collected by centrifugation (6000×G, 4°C). Supernatants were discarded and the protein pellet was frozen immediately at −80°C.

### Purification of MSP-1-BBM

Two antibody affinity columns were prepared by coupling approximately 5 mg of either mAb 12.2 or mAb 12.8, which recognize MSP-1 Block 2 and MSP-1_19_ respectively, to 5 mL Hi-Trap NHS-ester columns (GE Healthcare, UK) according to the manufacturer’s recommended protocol.

Ammonium sulfate precipitates from culture supernatants of *T. thermophila* expressing MSP-1-BBM were resuspended in Tris-buffered saline (25 mM Tris, pH 8.0, 100 mM NaCl). This solution was heated to 70°C for 20 minutes, cooled on ice for 20 minutes and finally centrifuged at 5000×G for 30 minutes. The feedstock was filtered using a 0.22 µM bottle-top filter (Pall Corporation, UK) then loaded onto the 5 mL 12.8 mAb affinity column and afterwards washed extensively with 25 mM Tris, pH 8.0, 100 mM NaCl. Bound proteins were eluted with 0.1 M Glycine, pH 2.7 into 1 M Tris, pH 9.0 neutralization buffer. The eluate was loaded onto the 12.2 mAb column, washed extensively with 25 mM Tris, pH 8.0, 100 mM NaCl and eluted with 0.1 M Glycine pH 2.7 into a 1 M Tris pH 9.0 neutralization buffer.

### Immunization of Mice

A group of five outbred female MF1 mice were immunized subcutaneously on days 0, 14 and 28 with 100 µL volumes containing 20 µg BBM protein formulated with CoVaccineHT (Protherics Medicines Development Limited, A BTG International Group Company, London, UK). The animals were exsanguinated on day 40 and serum prepared from each. Naïve mice from the same breeding stock were used to provide serum for negative control samples.

### Antigens and Antibody Titer Determination by ELISA

Sera from MSP-1-BBM immunized animals were tested by previously described ELISA for recognition of the MSP-1 Block 2 hybrid protein, MSP-1 Block 2 GST fusion proteins from all three Block 2 serotypes, and MSP-1_19_ GST fusion protein [31], [53], [54]. Negative control wells in ELISA were either coating buffer alone for the MSP-1 hybrid, or GST-coated wells for GST fusion proteins. All sera were tested across a range of tripling dilutions (1∶100 to 1∶218,700) against each antigen in duplicate wells, with a standard pool of Block 2- or MSP-1_19_- positive sera also tested on each plate, and across the same dilution range. Midpoint EC_50_ ELISA titers (arbitrary units) were calculated by interpolation from the fitted standard curve on each plate using polynomial logistic regression.

### Indirect Immunofluorescence Assays (IFA)

Serum samples from mice immunized with MSP-1-BBM were analyzed by IFA for parasite reactivity with the 3D7, MAD20 and RO33 isolates of *P. falciparum* by methods previously described [46], [54]. Endpoint titers were calculated as the highest dilution at which clear antibody reactivity with schizont stage parasites could be observed under FITC fluorescence. IFA results were scored independently by two experienced microscopists.

### ELISA with Biotinylated Peptides

A set of 133 biotinylated dodecapeptides covering all possible linear epitopes contained within MSP-1 Block 2 hybrid sequence were synthesized by Mimotopes Pyt. Ltd. (Clayton, Australia). ELISA plates (Greiner Bio One, UK) were coated with 100 µL of 5 µg mL^−1^ streptavidin (Sigma) and incubated at 37°C until dry. Plates were stored in heat sealed foil pouches with 1 g silica gel at room temperature until use. Reactivity of sera against the peptide library was determined by ELISA. Streptavidin-coated plates were washed in PBS-T (PBS, 0.05% Tween® 20) and blocked with blocking buffer (1% ByCoA, Croda Healthcare, UK dissolved in PBS) for 5 hours at room temperature. Peptide library plates were prepared by addition of 300 ng peptide per well, in duplicate, and plates were incubated overnight at 4°C. Sera were added to each well (100 µL at 1∶500 dilution) and incubated overnight at 4°C, then washed with PBS-T. Dilutions of a species-specific HRP-linked secondary antibody (Dako, UK), appropriate to the serum being tested, were added to each well and plates were incubated at room temperature for 3 hours. Plates were washed three times with PBS-T and OPD substrate was added to each well. Reactions were stopped by addition of 0.2 M sulfuric acid and absorbance was read at 492 nm using a microplate absorbance reader (Multiskan Ascent, Thermo Scientific, UK).

Background reactivity of serum antibodies with peptides was calculated as mean of all values in lowest two quartiles and a positive threshold for reactivity was set as background plus 4 times the standard deviation of these values. Antibody reactivity was categorized as high where the OD reading was greater than 1 above threshold, moderate if above threshold or negative when below threshold.

### Mass Spectrometry

Mass spectrometry services were provided by the BSRC Mass Spectrometry and Proteomics Facility, University of St Andrews. The purified MSP-1-BBM product was resolved on a 4–12% poly-acrylamide gel and the predominant gel band, of molecular weight of ∼60 kDa was excised and cut into 1 mm cubes. These were then subjected to in-gel digestion, using a ProGest Investigator in-gel digestion robot (Digilab) using standard protocols [45], [55], [56]. Briefly the gel cubes were destained by washing with a 1∶1 mixture of 30 mM potassium ferricyanide and 100 mM sodium thiosulphate and subjected to reduction and alkylation before digestion with trypsin at 37°C. The peptides were extracted with 10% formic acid and concentrated down using a SpeedVac (ThermoSavant).

The peptides were then separated on an Acclaim PepMap 100 C18 trap and an Acclaim PepMap RSLC C18 column (ThermoFisher Scientific), using a nanoLC Ultra 2D plus loading pump and nanoLC as-2 autosampler (Eskigent). The peptides were eluted with a gradient of increasing acetonitrile, containing 0.1% formic acid (5–40% acetonitrile in 5 min, 40–95% in a further 1 min, followed by 95% acetonitrile to clean the column, before reequilibration to 5% acetonitrile). The eluent was sprayed into a TripleTOF 5600 electrospray tandem mass spectrometer (ABSciex) and analysed in Information Dependent Acquisition (IDA) mode, performing 250 msec of MS followed by 100 msec MSMS analyses on the 20 most intense peaks seen by MS. The MS/MS data file generated was analysed using the Mascot algorithm (Matrix Science) against a modified version of the NCBInr database Aug 2013 incorporating the synthetic sequence of the MSP-1-BBM protein. Searches were performed with no species restriction, trypsin as the cleavage enzyme, carbamidomethyl as a fixed modification of cysteines and methionine oxidation and deamidation of glutamines and asparagines as a variable modifications.

## Results

### Construction of the MSP-1-BBM Synthetic Gene and Generation of Recombinant *T. thermophila*

**Figure 1:**
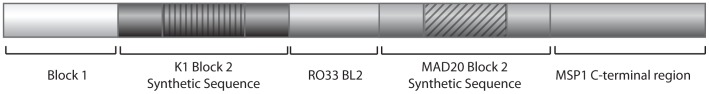
Schematic diagram of the MSP-1-BBM construct which encodes the N-terminal MSP-1 Block 1 region, the K1 Block 2 synthetic sequence, the RO33 Block 2 sequence, the MAD20 Block 2 synthetic sequence [31] and a C-terminal fragment encoding residues 1571 to 1702 of MSP-1 precursor protein of *P. falciparum*. The Block 2 region is arranged similar to natural alleles. Synthetic Block 2 repeat sequences of the K1 and MAD20 serotypes are indicated by vertical and diagonal hatched markings, respectively.

A synthetic vaccine construct was synthesized that comprised sequences from both conserved and polymorphic regions of MSP-1: MSP-1 Blocks 1, 2, 16 and 17. The Block 1 sequence and Block 2 synthetic hybrid sequence used was identical to that previously described for the MSP-1 hybrid antigen [18], [31]. To this, the highly conserved MSP-1_19_ sequence without its GPI anchor sequence was added C-terminally, in addition to an upstream 35 amino acid region from Block 16. The Block 16 sequence is thought to be important in dimerization of MSP-1 when it is in the MSP-1_42_ form prior to secondary proteolytic processing [45], [57]. The MSP-1 signal peptide (residues 1–19) sequence was added to enable secretion of the protein into the culture supernatant [18], [31]. The resulting construct, MSP-1-BBM (MSP-1 **B**lock 1, **B**lock 2, **M**SP-1_19_), is 498 amino acids in size and is schematically represented in Figure 1A.

The MSP 1-BBM DNA sequence was codon-optimized for the host’s native codon usage frequency. During gene synthesis, putative N-glycosylation sites encoded at MSP-1-BBM nucleotide positions 538–540 and 715–717 were removed by substitution of these asparagine residues with glutamine. The DNA sequence was synthesized commercially and was successfully assembled with cadmium inducible MTT1 promoter and the BTU2 terminator [53], [57] to create the final MSP-1-BBM expression cassette. The expression cassette was transferred to the integrative acceptor vector p22X that directed the transgene into the macronuclear genome of *T. thermophila* by homologous recombination and enabled selection of transformants by paromomycin.

Transformants were screened for expression and secretion of MSP-1-BBM in the presence and absence of the inducer cadmium chloride by SDS-PAGE and Western blot (data not shown). MSP-1-BBM protein was detected in cadmium-induced cultures and a single transformant with the highest secretion activity was selected for all further work. An isocitrate dehydrogenase (ICDH) assay indicated that MSP-1-BBM was present without the release of elevated levels of the cytoplasmic enzyme ICDH, confirming that extracellular MSP-1-BBM was not released upon cytolysis but instead derived from protein export (data not shown).

### Expression and Purification of MSP-1-BBM in *T. thermophila*

**Figure 2:**
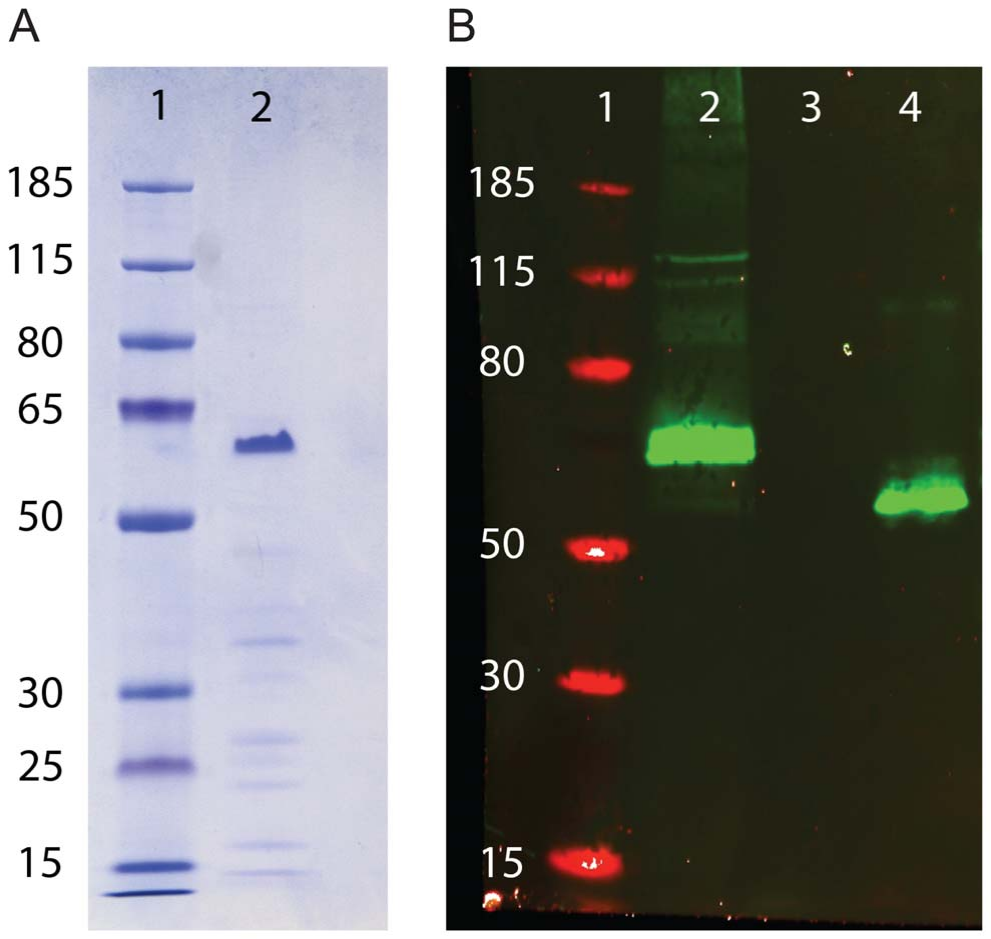
Analysis of purified MSP-1-BBM protein by SDS-PAGE and Western blotting. A. Coomassie-stained gel of purified MSP-1-BBM protein produced in *T. thermophila*. Lane 1. Molecular weight markers. Lane 2. 0.5 µg purified MSP-1-BBM protein. B. Western blot of MSP-1-BBM protein and MSP-1 hybrid probed with with mAb 12.2, (specific for repeat sequences present in the K1 serotype of MSP-1 Block 2). Lane 1. Molecular weight markers. Lane 2. 0.5 µg of *Tetrahymena*-derived MSP-1-BBM protein. Lane 3. Negative control. Lane 4. 0.5 µg MSP-1 Block 2 hybrid protein [30] (positive control).

The selected MSP-1-BBM *T. thermophila* cells were successfully cultured in a 50 L stirred cell bioreactor and expression was induced by addition of cadmium chloride. At the end of the batch fermentation run, culture broth was harvested and concentrated by precipitation with ammonium sulfate, yielding a wet pellet weight of 313 g. As MSP 1-BBM is a thermostable protein, the resuspended ammonium sulfate precipitate was heated to 70°C for 20 minutes, then centrifuged to remove contaminating denatured proteins. MSP 1-BBM protein was then purified from the supernatant of the heat-treated sample by affinity chromatography, using two different affinity columns. The first mAb affinity column (mAb 12.8) bound the C-terminal MSP-1_19_ portion of the protein. The eluate was loaded onto a second column coupled with mAb 12.2 to bind proteins containing the N-terminal Block 2 domain of MSP-1. The purified protein was analyzed on Coomassie stained SDS-PAGE gels and by Western blotting with mAb 12.2 (Figure 2, panels A and B). A dominant band of approximately 60 kDa was seen, corresponding to full-length recombinant MSP-1-BBM (calculated molecular weight: 48.3 kDa). A number of minor bands of lower molecular weight were present on the SDS-PAGE gel but not on the Western blot. In Western blot analysis the 12.2 mAb reacted specifically with a major band of approximately 60 kDa and with two minor bands of approximately 114 and 120 kDa. The final yield of MSP-1-BBM protein was determined by Bradford assay to be 0.5 mg.

The dominant ∼60 kDa gel band was excised from a silver stained SDS-PAGE gel and analyzed by electrospray tandem mass spectrometry. Database searching of identified peptide sequences against a custom NCBI database, which included the synthetic MSP-1-BBM sequence, confirmed the identity of the excised protein as MSP-1-BBM.

### Immunogenicity Testing of Purified MSP-1-BBM

**Figure 3:**
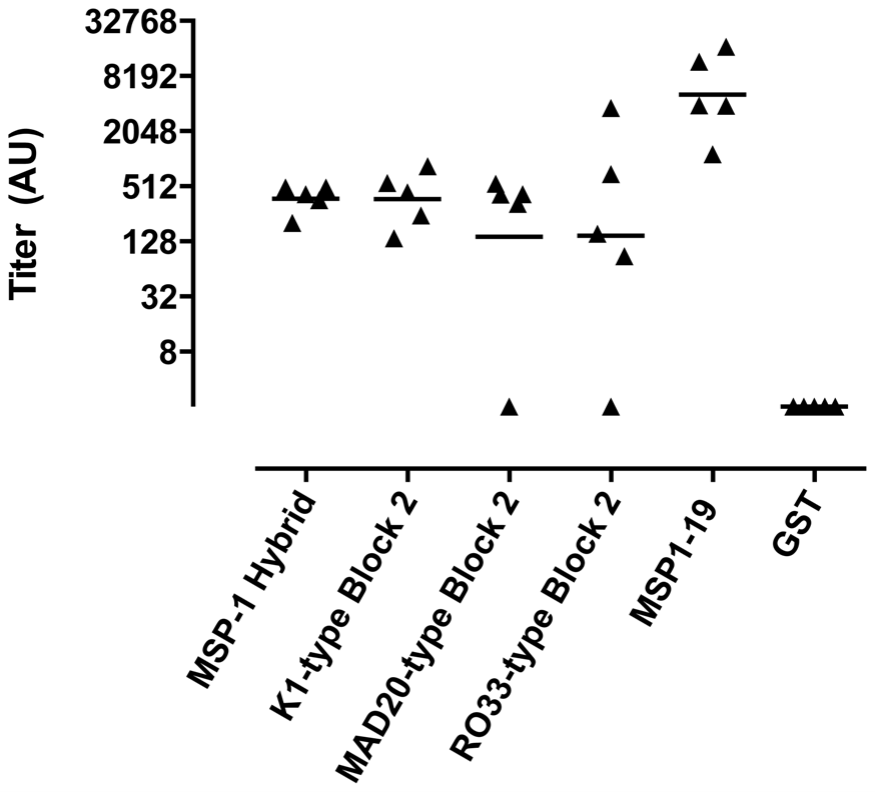
Immunogenicity in mice of recombinant MSP-1-BBM protein from *T. thermophila* [49]. A group of five MF1 mice were immunized s.c. three times, at 2 week intervals with MSP-1-BBM protein formulated in CoVaccine HT as described. Twelve days after the last immunization (d40), serum samples from each animal were tested by ELISA for antibody responses against the MSP-1 Block 2 hybrid protein [31] K1-type Block 2 protein [54], MAD20-type Block 2 protein [54], RO33-type Block 2 protein [54] and MSP-1_19_ protein [53]. Titers were calculated as outlined in materials and methods and expressed as arbitrary units (AU). Data is shown on a natural logarithmic scale as dotplots of serum reactivity for individual animals with the median level of Ab reactivity indicated by the solid horizontal line.

The immunogenicity of the MSP-1-BBM protein was tested by immunizing mice subcutaneously on days 0, 14 and 28, then generating sera from blood obtained by exsanguination on day 40. Sera from immunized mice and control sera from naïve mice were tested by ELISA against MSP-1 Block 2 hybrid protein, MSP-1 Block 2-GST fusion proteins from all three Block 2 serotypes and MSP-1_19_-GST fusion protein (Figure 3). Sera from all five MSP-1-BBM immunized mice reacted strongly with both the MSP-1 hybrid protein (geometric mean 376 AU, range 203 AU to 496 AU), K1-type Block 2 protein (geometric mean 371 AU, range 139 Au to 847 AU), MAD20-type Block2 protein (geometric mean 144 AU, range 2 AU to 544 AU), RO33-type Block 2 protein (geometric mean 144 AU, range 2 AU to 3651 AU) and MSP-1_19_-GST fusion protein (geometric mean 5112 AU, range 1148 AU to 11736 AU) but did not react with GST alone. Sera from naïve mice gave no increased response above background against any of the three antigens tested (data not shown).

### Immunofluorescence and Western Blotting

**Figure 4:**
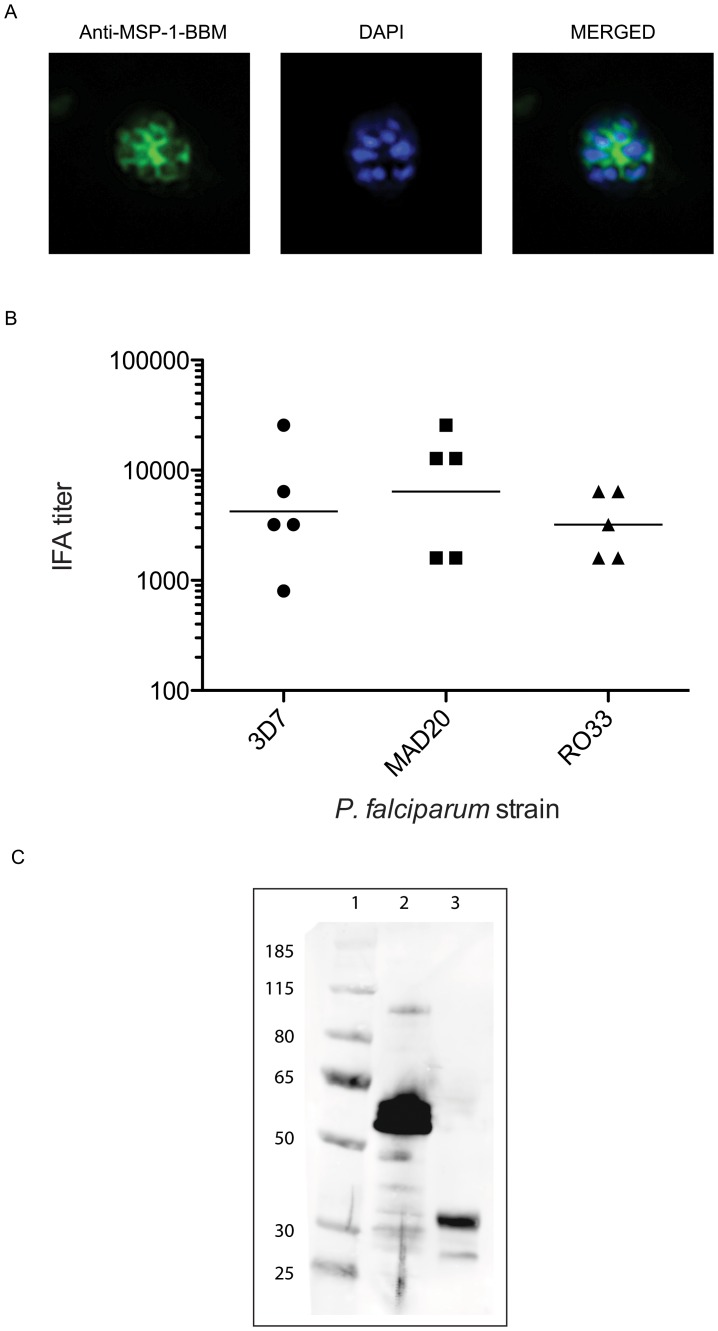
Indirect immunofluorescence assay (IFA) of sera from MSP-1-BBM immunized mice against three strains of *P. falciparum*. A. Representative micrograph of IFA assay with sera from MSP-1-BBM immunized mice. DAPI staining of parasite nuclei is shown in blue and fluorescence from the FITC-conjugated secondary antibody is shown in green. B. IFA titers of sera from mice immunized with MSP-1-BBM protein. Sera were tested by IFA against the 3D7 (K1 serotype), MAD20 and RO33 strains of *P. falciparum*, as described in materials and methods. IFA endpoint data is shown on a log_10_ scale on the Y axis. Each symbol represents the serum reactivity for an individual animal, with the geometric mean of Ab reactivity against each parasite strain indicated by the solid line. C. Western blot of MSP-1 Block 2 hybrid and MSP1_19_ proteins probed with pooled serum from mice immunized with MSP-1-BBM protein. Lane 1: Molecular weight markers, Lane 2: MSP-1 block 2 hybrid protein, Lane 3: MSP1_19_-GST fusion protein.

Sera were tested by immunofluorescence assay (IFA) for reactivity against *P. falciparum* blood stage parasites from three strains representative of each of the three MSP-1 Block 2 serotypes; 3D7, MAD20 and RO33 (Figure 4). High antibody titers were observed for all mice against each parasite strain. Sera reacted against parasites from ring, trophozoite and schizont stages, with strongest reactivity against the schizont stages where the antibody localized to the surface of the individual merozoites (Figure 4A). IFA endpoint titers were similar for all three strains of parasite (3D7 parasite strain geometric mean titer 4222, range 800–25,600; MAD20 strain geometric mean titer 6400, range 1600–25,600; RO33 strain geometric mean 3840, range 1600–6400) (Figure 4B). Differences between IFA titers were not statistically significant (Kruskal-Wallis test, p = 0.667).

To confirm the presence of both MSP-1 Block 1/Block 2 sequences and the MSP-1_19_ component within the MSP-1-BBM immunogen, a pool of serum from all MSP-1-BBM immunized mice was tested by Western blot against both the recombinant MSP-1 Block 2 hybrid protein [18], [19], [31], [58] and a MSP1_19_-GST fusion protein [18], [53], [59]. The pooled serum reacted strongly with both recombinant proteins, confirming that antibodies were elicited to both the C-terminal MSP-1_19_ region, and to the N-terminal Block1/2 part of MSP-1 (Figure 4C).

### Epitope Mapping by ELISA

**Figure 5:**
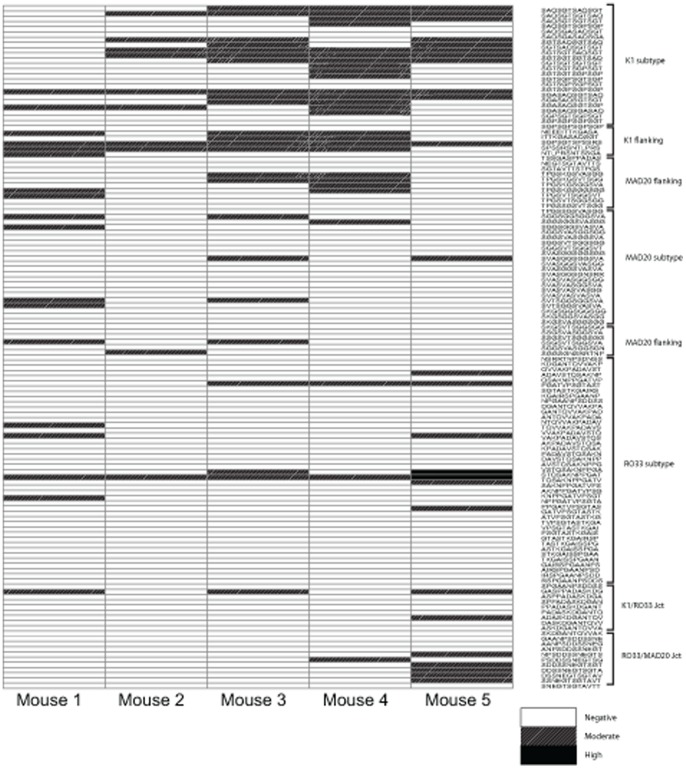
Epitope mapping of sera from MSP-1-BBM immunized mice by recognition of peptide epitopes within the MSP-1 Block 2 region of the MSP-1-BBM construct. A series of 133-terminally biotinylated dodecapeptides, representing the sequence diversity of all three Block 2 serotypes were used in ELISA to map the antibody specificities present in the sera of immunized animals. Reactivity with individual peptides is shown as shaded boxes, with the depth of shading of each box representing the strength of reactivity of a 1∶500 dilution of sera with each peptide. The sequences and Block 2 serotype (K1, MAD20 and RO33) of each peptide are indicated down the right hand side of the diagram.

To assess the epitope and serotype specificity of antibody responses against the Block 2 region of MSP-1, sera were tested by ELISA for reactivity with a panel of peptides containing all of the linear epitopes from the three serotypes of Block 2 (Figure 5). Antibody reactivity was observed against peptides containing epitopes from all three Block 2 serotypes, but greater reactivity was directed against peptides derived from the K1 Block 2 serotype than the MAD20 serotype or RO33 serotype (Mann-Whitney tests, p = 0.027 and p = 0.008 respectively).

## Discussion

In this study we have successfully demonstrated recombinant production of a malaria vaccine candidate antigen using the ciliate protozoan *T. thermophila*. This expression platform has previously been successfully used in a number of applications to express and secrete heterologous proteins of therapeutic interest, and boasts a number of favorable characteristics such as scalable growth to high cell densities, eukaryotic protein folding, ability to target protein for secretion and low biological hazard [18], [19], [58], [60], [61]. However, the production of *Plasmodium* proteins in *Tetrahymena* as potential vaccines has additional advantages, since *Tetrahymena* and *Plasmodium* are closely related and share some characteristics important for protein expression [17], [18], [59]. In particular, the prevalence of AT-rich genes and highly repetitive sequence content of *P. falciparum* can prove technically problematic for heterologous expression in other expression systems [60]–[63]. Previous studies have demonstrated that *T. thermophila* is able to express *Plasmodium* proteins [17], [18], [58], but we are currently unaware of any reports of the production of soluble malaria antigens using the *T. thermophila* expression host.

Whereas *P. falciparum* makes little or no use of post-translationally added N-linked glycans [62]–[64], *T. thermophila* extracellular proteins can be modified with a high-mannose N-glycosylation pattern [31], [58], [65], [66]. All potential N-glycosylation sites were removed from the synthetic gene to prevent inappropriate N-glycosylation of MSP-1-BBM, which can have deleterious effects on the magnitude and specificity of immune responses generated to immunogens [34]–[36], [64]. We assume that these modifications had no deleterious effect on the conformation of MSP-1-BBM because a strong antibody response against native parasite antigens was elicited by immunization with the purified construct.

We successfully achieved secretion of the MSP-1-BBM protein into the culture medium, as demonstrated by the presence of the protein in the absence of elevated levels of the cytoplasmic protein isocitrate dehydrogenase. This indicates that the MSP-1 signal peptide was processed by a *T. thermophila* specific endopeptidase, resulting in the release of the mature MSP-1-BBM antigen. While the synthetic Block 2 domain is an intrinsically unstructured protein domain, due to its unusual amino acid content [31], [65]–[67], MSP-1_19_ contains two cysteine-rich EGF-like motifs [34]–[36], [58], whose conformational integrity is required to elicit invasion inhibitory antibodies [57], [67]. The targeting of MSP-1-BBM via the secretory pathway ensured passage through the ER and Golgi apparatus giving optimal likelihood for correct folding and disulfide bond formation.

To produce MSP-1-BBM protein for immunogenicity testing, a selected stable expression strain was cultivated in a batch fermentation process at 50-liter scale. High cell densities of ∼1.7×10^6^ cells mL^−1^ were reached in a standard bioreactor (Biostat UD 50 fermentor, Sartorius, Goettingen, Germany) [58], [68], [69]. The cadmium-inducible *MTT1* promoter regulated recombinant antigen expression [57], [68], [70], so that timing of MSP-1-BBM expression could be modified to start in the early logarithmic phase of the cell culture. Western blot analysis revealed expression of extracellular MSP-1-BBM after cadmium addition and the soluble protein reacted with antibodies specific for either the synthetic MSP-1 Block 2 sequence (Figure 2B) or the MSP-1_19_ domain (data not shown).

Following trials of a number of biochemical purification techniques, protein purification was achieved using heat treatment followed by a simple two-step monoclonal-antibody affinity chromatography purification protocol. In the first step, mAb 12.8 that recognizes MSP-1_19_ was used [31], [68], [69]. In the second step, mAb 12.2 that recognizes an MSP-1 Block 2 epitope was used to further purify MSP-1-BBM. The 12.8 mAb binds to a highly conformational epitope [68], [70]–[72], therefore we expect that this step would purify only correctly folded protein. Use of a second affinity column (mAb 12.2) favored the purification of full-length protein, as both the Block 2 and Block 17 regions were selected for during purification. A heat step had previously been shown to be effective for the purification of the MSP-1 hybrid antigen [31], [60], in addition to the MSP-2 and MSP-3 proteins [34], [71], [72]. These proteins are resistant to heat-induced protein aggregation, due to the intrinsically unstructured nature of these predominantly hydrophilic proteins. It was surprising to observe that the MSP-1-BBM protein also exhibited heat stability, despite it containing a substantial proportion of hydrophobic residues and including a highly conformationally defined region (MSP-1_19_) comprised of two EGF-like motifs [31], [60]. However, these motifs are highly stabilized by the presence of six disulphide bridges [34], [73] which may provide sufficient structural stability to prevent denaturation and aggregation of this protein moiety. The thermostability of an antigen not only facilitates purification but also is advantageous in terms of malaria vaccine development by making a vaccine cold chain unnecessary [31], [74].

After the heat step and affinity purification, 0.5 mg of full-length MSP-1-BBM was obtained. Currently, no extensive optimization of the production process has been carried out. Further gains in production efficiency can be made through clone screening or by optimization of harvesting and purification protocols, which would have been facilitated by the use of affinity-tags or fusions to enzymes such as glutathione-S-transferase (GST) [65], [73]. Since the main object of this project was to express a vaccine antigen, we avoided the use of heterologous tags or fusions that may have detrimentally influenced the immunogenicity or antigenicity of the malaria vaccine antigen, as has been observed before [31], [74]. We anticipate that this further work could achieve greater yields of this artificial and intrinsically unstructured antigen.

Purified MSP-1-BBM protein resolved as a major band in SDS-PAGE corresponding to a molecular weight of approximately 60 kDa, whereas the predicted molecular weight from primary sequence was 48.3 kDa. This slow migration was anticipated since the MSP-1 Block 2 hybrid component has previously been shown to exhibit slower electrophoretic mobility, as this predominantly hydrophilic amino acid containing region of MSP-1-BBM binds SDS weakly. As proteins with intrinsically unstructured regions often have slow electrophoretic mobility [31], we confirmed the identity of the 60 kDa band as MSP-1-BBM by tandem mass spectrometry (see Materials and Methods). Some minor bands of 10–45 kDa were observed, and may indicate slight contamination with host cell proteins or the presence of degradation products, which were not detected by the 12.2 mAb, which detects an N-terminal MSP-1 epitope in MSP-1-BBM. A minor band of approximately 120 kDa was observed on Western blots, which is likely to be a dimeric form of MSP-1-BBM, although it is unknown whether the putative dimerization region of Block 16 that is present in the MSP-1-BBM construct mediates this.

The MSP-1-BBM protein was immunogenic in mice when formulated with CoVaccineHT, raising high titers of antibodies against both the MSP-1 hybrid and MSP-1_19_ components, including responses against all three MSP-1 block 2 serotypes (Figures 3 and 4C). This confirms that the resulting construct was both immunogenic using an adjuvant potentially suitable for human use and antigenically similar to the MSP-1 hybrid. The resulting antibodies reacted very strongly with parasites of all three serotypes tested as assessed by IFA (Figure 4A), providing evidence that the recombinant protein is antigenically similar to native parasite proteins. As shown in Figure 5, antibody responses were demonstrated to react with a number of individual peptides composed of epitopes from the three Block 2 serotypes. However, there was greater recognition of peptides containing epitopes from the K1 type Block 2 serotype than the MAD20 or RO33 Block 2 serotypes. Murine humoral responses are often more oligoclonal than other species, and recent pre-clinical validation of the related MSP-1 hybrid vaccine antigen showed that rabbits and monkeys produced broader antibody responses to a larger number of epitopes within the MSP-1 hybrid protein [31], [49]. In addition, peptide ELISA is intrinsically less sensitive than ELISA using whole proteins, so weaker responses may have been undetected in this peptide-based ELISA. It is clear from the results of immunofluorescence assays that antibodies to all three MSP-1 Block 2 serotypes were produced by all immunized mice, which indicates that the MSP-1-BBM antigen can elicit a broad, strain transcending antibody response to multiple parasite serotypes.

Sera from immunized mice reacted with parasites from ring, trophozoite and schizont parasite stages. The Block 1, 2 and 16 components would be expected to elicit antibodies that react with parasite stages expressing full-length MSP-1 (i.e. schizonts) in IFA compared with antibodies to MSP-1_19_, which would react with all blood stages. This is explained by the fact that protein regions coded by blocks 1, 2 and 16 are shed along with the remainder of the MSP-1 complex upon merozoite invasion, leaving only the Block 17 (MSP-1_19_) component on the cell surface. This is likely to account for the much brighter appearance of parasites of the schizont stage in IFA, where the antibodies to all MSP-1 components can bind parasites, compared to the more selective, MSP-1_19_ specific reactivity seen with trophozoites and rings. To confirm the specificity of the antibodies raised by immunization with MSP-1-BBM, we showed that the sera from immunized mice reacted strongly with both the MSP-1 Block 2 hybrid protein and MSP1_19_-GST fusion protein in Western blot, confirming that antibodies were successfully raised against both individual components of the vaccine antigen.

This fusion protein was evaluated with the aim of further development as a malaria blood-stage vaccine, but there are internal proteolytic cleavage sites in the vaccine antigen, separating the MSP-1_19_ component from the MSP-1 Block1/2 component, which led to reduced overall yields of full-length MSP-1-BBM. This would complicate reproducible production of the protein under cGMP. Future work will involve the removal of these proteolytically sensitive sites by genetic manipulation of the gene, and improvement of fermentation conditions using protease knockout *T. thermophila* strains.

## Conclusion

This study has successfully demonstrated the expression and purification of a promising malaria vaccine candidate antigen in the *Tetrahymena thermophila* expression system. We examined the immunological characteristics of MSP-1-BBM secreted from *T. thermophila* cells in MF1 mice and demonstrated that antibodies raised to the protein showed reactivity with MSP-1 protein epitopes from multiple parasite serotypes. The vaccine antigen proved immunogenic using an adjuvant suitable for use in humans and elicited antibodies in mice that recognized native parasite antigens from all three MSP-1 Block 2 serotypes. Altogether, this study represents an important step towards the establishment of the *Tetrahymena* expression system for malaria antigens that will provide a valuable tool for researchers facing the challenges that some malaria antigens can present in more conventional expression systems.

## References

[1] WHO (2011) Questions and Answers on Malaria Vaccine. Available: http://www.who.int/vaccine_research/diseases/malaria/malaria_vaccine_questions_and_answers_october_2011.pdf. Accessed 2013 Jun 3.

[2] malERA Consultative Group on Vaccines (2011) A research agenda for malaria eradication: vaccines. 8: e1000398 Available: http://eutils.ncbi.nlm.nih.gov/entrez/eutils/elink.fcgi?dbfrom=pubmed&id=21311586&retmode=ref&cmd=prlinks. 10.1371/journal.pmed.1000398 PMC3026701 21311586

[3] Hill AVS (2011) Vaccines against malaria. Philosophical transactions of the Royal Society of London Series B, Biological sciences 366: 2806–2814 Available: http://eutils.ncbi.nlm.nih.gov/entrez/eutils/elink.fcgi?dbfrom=pubmed&id=21893544&retmode=ref&cmd=prlinks. 21893544 10.1098/rstb.2011.0091 PMC3146776

[4] Crompton PD, Pierce SK, Miller LH (2010) Advances and challenges in malaria vaccine development. The Journal of clinical investigation 120: 4168–4178 Available: http://eutils.ncbi.nlm.nih.gov/entrez/eutils/elink.fcgi?dbfrom=pubmed&id=21123952&retmode=ref&cmd=prlinks. 21123952 10.1172/JCI44423 PMC2994342

[5] Huckriede A, Bungener L, Veer ter W, Holtrop M, Daemen T, et al (2003) Influenza virosomes: combining optimal presentation of hemagglutinin with immunopotentiating activity. Vaccine 21: 925–931 Available: http://eutils.ncbi.nlm.nih.gov/entrez/eutils/elink.fcgi?dbfrom=pubmed&id=12547604&retmode=ref&cmd=prlinks. 12547604 10.1016/s0264-410x(02)00542-x

[6] Milich DR, Hughes J, Jones J, Sällberg M, Phillips TR (2001) Conversion of poorly immunogenic malaria repeat sequences into a highly immunogenic vaccine candidate. Vaccine 20: 771–788 Available: http://eutils.ncbi.nlm.nih.gov/entrez/eutils/elink.fcgi?dbfrom=pubmed&id=11738741&retmode=ref&cmd=prlinks. 11738741 10.1016/s0264-410x(01)00400-5

[7] Victor ME, Bengtsson A, Andersen G, Bengtsson D, Lusingu JP, et al (2010) Insect cells are superior to Escherichia coli in producing malaria proteins inducing IgG targeting PfEMP1 on infected erythrocytes. Malaria Journal 9: 325 Available: http://eutils.ncbi.nlm.nih.gov/entrez/eutils/elink.fcgi?dbfrom=pubmed&id=21078147&retmode=ref&cmd=prlinks. 21078147 10.1186/1475-2875-9-325 PMC2994891

[8] Stowers AW, Chen L-H, Zhang Y, Kennedy MC, Zou L, et al (2002) A recombinant vaccine expressed in the milk of transgenic mice protects Aotus monkeys from a lethal challenge with Plasmodium falciparum. Proceedings of the National Academy of Sciences of the United States of America 99: 339–344 Available: http://eutils.ncbi.nlm.nih.gov/entrez/eutils/elink.fcgi?dbfrom=pubmed&id=11752405&retmode=ref&cmd=prlinks. 11752405 10.1073/pnas.012590199 PMC117562

[9] Lau OS, Ng DW-K, Chan WWL, Chang SP, Sun SSM (2010) Production of the 42-kDa fragment of Plasmodium falciparum merozoite surface protein 1, a leading malaria vaccine antigen, in Arabidopsis thaliana seeds. Plant biotechnology journal 8: 994–1004 Available: http://eutils.ncbi.nlm.nih.gov/entrez/eutils/elink.fcgi?dbfrom=pubmed&id=20444208&retmode=ref&cmd=prlinks. 20444208 10.1111/j.1467-7652.2010.00526.x

[10] Pollack Y, Katzen AL, Spira DT, Golenser J (1982) The genome of Plasmodium falciparum. I: DNA base composition. 10: 539–546 Available: http://eutils.ncbi.nlm.nih.gov/entrez/eutils/elink.fcgi?dbfrom=pubmed&id=6278419&retmode=ref&cmd=prlinks. 10.1093/nar/10.2.539 PMC326156 6278419

[11] Gardner MJ, Hall N, Fung E, White O, Berriman M, et al (2002) Genome sequence of the human malaria parasite Plasmodium falciparum. Nature 419: 498–511 Available: http://eutils.ncbi.nlm.nih.gov/entrez/eutils/elink.fcgi?dbfrom=pubmed&id=12368864&retmode=ref&cmd=prlinks. 12368864 10.1038/nature01097 PMC3836256

[12] Eisen JA, Coyne RS, Wu M, Wu D, Thiagarajan M, et al (2006) Macronuclear genome sequence of the ciliate Tetrahymena thermophila, a model eukaryote. 4: e286 Available: http://biology.plosjournals.org/perlserv/?request=get-document&doi=10.1371/journal.pbio.0040286&ct=1. 10.1371/journal.pbio.0040286 PMC1557398 16933976

[13] Hellenbroich D, Valley U, Ryll T, Wagner R, Tekkanat N, et al (1999) Cultivation of Tetrahymena thermophila in a 1.5-m3 airlift bioreactor. 51: 447–455 Available: http://eutils.ncbi.nlm.nih.gov/entrez/eutils/elink.fcgi?dbfrom=pubmed&id=10341428&retmode=ref&cmd=prlinks. 10.1007/s002530051415 10341428

[14] Kiy T, Tiedtke A (1992) Continuous high-cell-density fermentation of the ciliated protozoon Tetrahymena in a perfused bioreactor. 38: 141–146 Available: http://eutils.ncbi.nlm.nih.gov/entrez/eutils/elink.fcgi?dbfrom=pubmed&id=1369137&retmode=ref&cmd=prlinks. 10.1007/BF00174458 1369137

[15] Jayaram J, Papoyan A, Bisharyan Y, Cassidy-Hanley D, Zhang X, et al. (2010) An Alternative Platform for Rapid Production of Effective Subunit Vaccines. BioPharm International Supplements. Available: http://www.biopharminternational.com/biopharm/Vaccine+Manufacturing+Articles/An-Alternative-Platform-for-Rapid-Production-of-Ef/ArticleStandard/Article/detail/690797.

[16] Gaertig J, Gao Y, Tishgarten T, Clark TG, Dickerson HW (1999) Surface display of a parasite antigen in the ciliate Tetrahymena thermophila. Nature Biotechnology 17: 462–465 Available: http://eutils.ncbi.nlm.nih.gov/entrez/eutils/elink.fcgi?dbfrom=pubmed&id=10331805&retmode=ref&cmd=prlinks. 10.1038/8638 10331805

[17] Peterson DS, Gao Y, Asokan K, Gaertig J (2002) The circumsporozoite protein of Plasmodium falciparum is expressed and localized to the cell surface in the free-living ciliate Tetrahymena thermophila. Molecular and biochemical parasitology 122: 119–126 Available: http://eutils.ncbi.nlm.nih.gov/entrez/eutils/elink.fcgi?dbfrom=pubmed&id=12106865&retmode=ref&cmd=prlinks. 12106865 10.1016/s0166-6851(02)00079-8

[18] Weide T, Bockau U, Rave A, Herrmann L, Hartmann MWW (2007) A recombinase system facilitates cloning of expression cassettes in the ciliate Tetrahymena thermophila. BMC microbiology 7: 12 Available: http://eutils.ncbi.nlm.nih.gov/entrez/eutils/elink.fcgi?dbfrom=pubmed&id=17328820&retmode=ref&cmd=prlinks. 17328820 10.1186/1471-2180-7-12 PMC1839094

[19] Aldag I, Bockau U, Rossdorf J, Laarmann S, Raaben W, et al (2011) Expression, secretion and surface display of a human alkaline phosphatase by the ciliate Tetrahymena thermophila. BMC biotechnology 11: 11 Available: http://eutils.ncbi.nlm.nih.gov/entrez/eutils/elink.fcgi?dbfrom=pubmed&id=21281462&retmode=ref&cmd=prlinks. 21281462 10.1186/1472-6750-11-11 PMC3042934

[20] Sanders PR, Gilson PR, Cantin GT, Greenbaum DC, Nebl T, et al (2005) Distinct protein classes including novel merozoite surface antigens in Raft-like membranes of Plasmodium falciparum. The Journal of biological chemistry 280: 40169–40176 Available: http://eutils.ncbi.nlm.nih.gov/entrez/eutils/elink.fcgi?dbfrom=pubmed&id=16203726&retmode=ref&cmd=prlinks. 16203726 10.1074/jbc.M509631200

[21] Sanders PR, Cantin GT, Greenbaum DC, Gilson PR, Nebl T, et al (2007) Identification of protein complexes in detergent-resistant membranes of Plasmodium falciparum schizonts. Molecular and biochemical parasitology 154: 148–157 Available: http://eutils.ncbi.nlm.nih.gov/entrez/eutils/elink.fcgi?dbfrom=pubmed&id=17553576&retmode=ref&cmd=prlinks. 17553576 10.1016/j.molbiopara.2007.04.013

[22] Tanabe K, Mackay M, Goman M, Scaife JG (1987) Allelic dimorphism in a surface antigen gene of the malaria parasite Plasmodium falciparum. Journal of molecular biology 195: 273–287 Available: http://linkinghub.elsevier.com/retrieve/pii/0022283687906498. 3079521 10.1016/0022-2836(87)90649-8

[23] Jiang G, Daubenberger C, Huber W, Matile H, Tanner M, et al (2000) Sequence diversity of the merozoite surface protein 1 of Plasmodium falciparum in clinical isolates from the Kilombero District, Tanzania. Acta Tropica 74: 51–61 Available: http://eutils.ncbi.nlm.nih.gov/entrez/eutils/elink.fcgi?dbfrom=pubmed&id=10643908&retmode=ref&cmd=prlinks. 10643908 10.1016/s0001-706x(99)00045-5

[24] Miller LH, Roberts T, Shahabuddin M, McCutchan TF (1993) Analysis of sequence diversity in the Plasmodium falciparum merozoite surface protein-1 (MSP-1). Molecular and biochemical parasitology 59: 1–14 Available: http://eutils.ncbi.nlm.nih.gov/entrez/eutils/elink.fcgi?dbfrom=pubmed&id=8515771&retmode=ref&cmd=prlinks. 8515771 10.1016/0166-6851(93)90002-f

[25] Cavanagh DR, Dodoo D, Hviid L, Kurtzhals JAL, Theander TG, et al (2004) Antibodies to the N-terminal block 2 of Plasmodium falciparum merozoite surface protein 1 are associated with protection against clinical malaria. Infection and immunity 72: 6492–6502 Available: http://eutils.ncbi.nlm.nih.gov/entrez/eutils/elink.fcgi?dbfrom=pubmed&id=15501780&retmode=ref&cmd=prlinks. 15501780 10.1128/IAI.72.11.6492-6502.2004 PMC522997

[26] Mawili-Mboumba DP, Borrmann S, Cavanagh DR, McBride JS, Matsiegui P-B, et al (2003) Antibody responses to Plasmodium falciparum merozoite surface protein-1 and efficacy of amodiaquine in Gabonese children with P. falciparum malaria. J Infect Dis 187: 1137–1141 Available: http://eutils.ncbi.nlm.nih.gov/entrez/eutils/elink.fcgi?dbfrom=pubmed&id=12660928&retmode=ref&cmd=prlinks. 12660928 10.1086/368414

[27] Stanisic DI, Richards JS, McCallum FJ, Michon P, King CL, et al (2009) Immunoglobulin G Subclass-Specific Responses against Plasmodium falciparum Merozoite Antigens Are Associated with Control of Parasitemia and Protection from Symptomatic Illness. Infection and immunity 77: 1165–1174 Available: http://iai.asm.org/cgi/doi/10.1128/IAI.01129-08. 19139189 10.1128/IAI.01129-08 PMC2643653

[28] Conway DJ, Cavanagh DR, Tanabe K, Roper C, Mikes ZS, et al (2000) A principal target of human immunity to malaria identified by molecular population genetic and immunological analyses. Nat Med 6: 689–692 Available: http://eutils.ncbi.nlm.nih.gov/entrez/eutils/elink.fcgi?dbfrom=pubmed&id=10835687&retmode=ref&cmd=prlinks. 10835687 10.1038/76272

[29] Polley SD, Tetteh KKA, Cavanagh DR, Pearce RJ, Lloyd JM, et al (2003) Repeat sequences in block 2 of Plasmodium falciparum merozoite surface protein 1 are targets of antibodies associated with protection from malaria. Infection and immunity 71: 1833–1842 Available: http://eutils.ncbi.nlm.nih.gov/entrez/eutils/elink.fcgi?dbfrom=pubmed&id=12654798&retmode=ref&cmd=prlinks. 12654798 10.1128/IAI.71.4.1833-1842.2003 PMC152097

[30] Galamo CD, Jafarshad A, Blanc C, Druilhe P (2009) Anti–MSP1 Block 2 Antibodies Are Effective at Parasite Killing in an Allele-Specific Manner by Monocyte-Mediated Antibody-Dependent Cellular Inhibition. J Infect Dis 199: 1151–1154 Available: http://www.journals.uchicago.edu/doi/abs/10.1086/597426. 19284307 10.1086/597426

[31] Cowan GJM, Creasey AM, Dhanasarnsombut K, Thomas AW, Remarque EJ, et al (2011) A malaria vaccine based on the polymorphic block 2 region of MSP-1 that elicits a broad serotype-spanning immune response. PLoS ONE 6: e26616 doi:10.1371/journal.pone.0026616 22073118 10.1371/journal.pone.0026616 PMC3202563

[32] Quakyi IA, Currier J, Fell A, Taylor DW, Roberts T, et al (1994) Analysis of human T cell clones specific for conserved peptide sequences within malaria proteins. Paucity of clones responsive to intact parasites. Journal of immunology (Baltimore, Md : 1950) 153: 2082–2092 Available: http://eutils.ncbi.nlm.nih.gov/entrez/eutils/elink.fcgi?dbfrom=pubmed&id=8051413&retmode=ref&cmd=prlinks. 8051413

[33] Parra M, Hui G, Johnson AH, Berzofsky JA, Roberts T, et al (2000) Characterization of conserved T- and B-cell epitopes in Plasmodium falciparum major merozoite surface protein 1. Infection and immunity 68: 2685–2691 Available: http://eutils.ncbi.nlm.nih.gov/entrez/eutils/elink.fcgi?dbfrom=pubmed&id=10768960&retmode=ref&cmd=prlinks. 10768960 10.1128/iai.68.5.2685-2691.2000 PMC97475

[34] Chitarra V, Holm I, Bentley GA, Pêtres S, Longacre S (1999) The crystal structure of C-terminal merozoite surface protein 1 at 1.8 A resolution, a highly protective malaria vaccine candidate. Molecular cell 3: 457–464 Available: http://eutils.ncbi.nlm.nih.gov/entrez/eutils/elink.fcgi?dbfrom=pubmed&id=10230398&retmode=ref&cmd=prlinks. 10230398 10.1016/s1097-2765(00)80473-6

[35] Pizarro JC, Chitarra V, Verger D, Holm I, Pêtres S, et al (2003) Crystal structure of a Fab complex formed with PfMSP1–19, the C-terminal fragment of merozoite surface protein 1 from Plasmodium falciparum: a malaria vaccine candidate. Journal of molecular biology 328: 1091–1103 Available: http://eutils.ncbi.nlm.nih.gov/entrez/eutils/elink.fcgi?dbfrom=pubmed&id=12729744&retmode=ref&cmd=prlinks. 12729744 10.1016/s0022-2836(03)00376-0

[36] Morgan WD, Birdsall B, Frenkiel TA, Gradwell MG, Burghaus PA, et al (1999) Solution structure of an EGF module pair from the Plasmodium falciparum merozoite surface protein 1. Journal of molecular biology 289: 113–122 Available: http://eutils.ncbi.nlm.nih.gov/entrez/eutils/elink.fcgi?dbfrom=pubmed&id=10339410&retmode=ref&cmd=prlinks. 10339410 10.1006/jmbi.1999.2753

[37] Gaur D, Mayer D, Miller L (2004) Parasite ligand-host receptor interactions during invasion of erythrocytes by merozoites. International Journal for Parasitology 34: 1413–1429 Available: http://linkinghub.elsevier.com/retrieve/pii/S0020751904002152. 15582519 10.1016/j.ijpara.2004.10.010

[38] Moss DK, Remarque EJ, Faber BW, Cavanagh DR, Arnot DE, et al (2012) Plasmodium falciparum 19-Kilodalton Merozoite Surface Protein 1 (MSP1)-Specific Antibodies That Interfere with Parasite Growth In Vitro Can Inhibit MSP1 Processing, Merozoite Invasion, and Intracellular Parasite Development. Infection and immunity 80: 1280–1287 Available: http://iai.asm.org/cgi/doi/10.1128/IAI.05887-11. 22202121 10.1128/IAI.05887-11 PMC3294643

[39] Blackman MJ (1994) Antibodies inhibit the protease-mediated processing of a malaria merozoite surface protein. Journal of Experimental Medicine 180: 389–393 Available: http://www.jem.org/cgi/doi/10.1084/jem.180.1.389. 7516416 10.1084/jem.180.1.389 PMC2191569

[40] Egan AF, Burghaus P, Druilhe P, Holder AA, Riley EM (1999) Human antibodies to the 19 kDa C-terminal fragment of Plasmodium falciparum merozoite surface protein 1 inhibit parasite growth in vitro. Parasite immunology 21: 133–139 Available: http://eutils.ncbi.nlm.nih.gov/entrez/eutils/elink.fcgi?dbfrom=pubmed&id=10205793&retmode=ref&cmd=prlinks. 10205793 10.1046/j.1365-3024.1999.00209.x

[41] Dodoo D, Aikins A, Asamoah Kusi K, Lamptey H, Remarque E, et al (2008) Cohort study of the association of antibody levels to AMA1, MSP119, MSP3 and GLURP with protection from clinical malaria in Ghanaian children. Malaria Journal 7: 142 Available: http://www.malariajournal.com/content/7/1/142. 18664257 10.1186/1475-2875-7-142 PMC2529305

[42] Egan AF, Morris J, Barnish G, Allen S, Greenwood BM, et al (1996) Clinical immunity to Plasmodium falciparum malaria is associated with serum antibodies to the 19-kDa C-terminal fragment of the merozoite surface antigen, PfMSP-1. J Infect Dis 173: 765–769 Available: http://eutils.ncbi.nlm.nih.gov/entrez/eutils/elink.fcgi?dbfrom=pubmed&id=8627050&retmode=ref&cmd=prlinks. 8627050 10.1093/infdis/173.3.765

[43] Keitel WA, Kester KE, Atmar RL, White AC, Bond NH, et al (1999) Phase I trial of two recombinant vaccines containing the 19kd carboxy terminal fragment of Plasmodium falciparum merozoite surface protein 1 (msp-1(19)) and T helper epitopes of tetanus toxoid. Vaccine 18: 531–539 Available: http://eutils.ncbi.nlm.nih.gov/entrez/eutils/elink.fcgi?dbfrom=pubmed&id=10519944&retmode=ref&cmd=prlinks. 10519944 10.1016/s0264-410x(99)00221-2

[44] Ockenhouse CF, Angov E, Kester KE, Diggs C, Soisson L, et al (2006) Phase I safety and immunogenicity trial of FMP1/AS02A, a Plasmodium falciparum MSP-1 asexual blood stage vaccine. Vaccine 24: 3009–3017 Available: http://eutils.ncbi.nlm.nih.gov/entrez/eutils/elink.fcgi?dbfrom=pubmed&id=16356603&retmode=ref&cmd=prlinks. 16356603 10.1016/j.vaccine.2005.11.028

[45] Babon JJ, Morgan WD, Kelly G, Eccleston JF, Feeney J, et al (2007) Structural studies on Plasmodium vivax merozoite surface protein-1. Molecular and biochemical parasitology 153: 31–40 Available: http://eutils.ncbi.nlm.nih.gov/entrez/eutils/elink.fcgi?dbfrom=pubmed&id=17343930&retmode=ref&cmd=prlinks. 17343930 10.1016/j.molbiopara.2007.01.015

[46] Arnot DE, Cavanagh DR, Remarque EJ, Creasey AM, Sowa MPK, et al (2008) Comparative testing of six antigen-based malaria vaccine candidates directed toward merozoite-stage Plasmodium falciparum. Clinical and vaccine immunology : CVI 15: 1345–1355 Available: http://eutils.ncbi.nlm.nih.gov/entrez/eutils/elink.fcgi?dbfrom=pubmed&id=18550731&retmode=ref&cmd=prlinks. 18550731 10.1128/CVI.00172-08 PMC2546674

[47] Sauer B (1987) Functional expression of the cre-lox site-specific recombination system in the yeast Saccharomyces cerevisiae. Molecular and cellular biology 7: 2087–2096 Available: http://eutils.ncbi.nlm.nih.gov/entrez/eutils/elink.fcgi?dbfrom=pubmed&id=3037344&retmode=ref&cmd=prlinks. 3037344 10.1128/mcb.7.6.2087-2096.1987 PMC365329

[48] Gaertig JJ, Gu LL, Hai BB, Gorovsky MAM (1994) High frequency vector-mediated transformation and gene replacement in Tetrahymena. Nucleic acids research 22: 5391–5398 Available: http://eutils.ncbi.nlm.nih.gov/entrez/eutils/elink.fcgi?dbfrom=pubmed&id=7816630&retmode=ref&cmd=prlinks. 7816630 10.1093/nar/22.24.5391 PMC332088

[49] Cassidy-Hanley D, Bowen J, Lee JH, Cole E, VerPlank LA, et al (1997) Germline and somatic transformation of mating Tetrahymena thermophila by particle bombardment. Genetics 146: 135–147 Available: http://www.genetics.org/cgi/reprint/146/1/135. 9136007 10.1093/genetics/146.1.135 PMC1207932

[50] Gaertig J, Kapler G (2000) Transient and stable DNA transformation of Tetrahymena thermophila by electroporation. 62: 485–500 Available: http://www.ncbi.nlm.nih.gov/pubmed/10503213?dopt=abstract. 10.1016/s0091-679x(08)61552-6 10503213

[51] Laemmli UK (1970) Cleavage of structural proteins during the assembly of the head of bacteriophage T4. Nature 227: 680–685 Available: http://eutils.ncbi.nlm.nih.gov/entrez/eutils/elink.fcgi?dbfrom=pubmed&id=5432063&retmode=ref&cmd=prlinks. 5432063 10.1038/227680a0

[52] McBride JS, Newbold CI, Anand R (1985) Polymorphism of a high molecular weight schizont antigen of the human malaria parasite Plasmodium falciparum. The Journal of experimental medicine 161: 160–180 Available: http://eutils.ncbi.nlm.nih.gov/entrez/eutils/elink.fcgi?dbfrom=pubmed&id=2578540&retmode=ref&cmd=prlinks. 2578540 10.1084/jem.161.1.160 PMC2187544

[53] Burghaus PA, Holder AA (1994) Expression of the 19-kilodalton carboxy-terminal fragment of the Plasmodium falciparum merozoite surface protein-1 in Escherichia coli as a correctly folded protein. Molecular and biochemical parasitology 64: 165–169 Available: http://eutils.ncbi.nlm.nih.gov/entrez/eutils/elink.fcgi?dbfrom=pubmed&id=8078519&retmode=ref&cmd=prlinks. 8078519 10.1016/0166-6851(94)90144-9

[54] Cavanagh DR, McBride JS (1997) Antigenicity of recombinant proteins derived from Plasmodium falciparum merozoite surface protein 1. Molecular and biochemical parasitology 85: 197–211 Available: http://eutils.ncbi.nlm.nih.gov/entrez/eutils/elink.fcgi?dbfrom=pubmed&id=9106193&retmode=ref&cmd=prlinks. 9106193 10.1016/s0166-6851(96)02826-5

[55] Gharahdaghi F, Weinberg CR, Meagher DA, Imai BS, Mische SM (1999) Mass spectrometric identification of proteins from silver-stained polyacrylamide gel: a method for the removal of silver ions to enhance sensitivity. Electrophoresis 20: 601–605 doi:;10.1002/(SICI)1522-2683(19990301)20:3<601::AID-ELPS601>3.0.CO;2-6 10217175 10.1002/(SICI)1522-2683(19990301)20:3<601::AID-ELPS601>3.0.CO;2-6

[56] Shevchenko A, Wilm M, Vorm O, Mann M (1996) Mass spectrometric sequencing of proteins silver-stained polyacrylamide gels. Anal Chem 68: 850–858. 8779443 10.1021/ac950914h

[57] Shang Y, Song X, Bowen J, Corstanje R, Gao Y, et al (2002) A robust inducible-repressible promoter greatly facilitates gene knockouts, conditional expression, and overexpression of homologous and heterologous genes in Tetrahymena thermophila. Proceedings of the National Academy of Sciences of the United States of America 99: 3734–3739 Available: http://eutils.ncbi.nlm.nih.gov/entrez/eutils/elink.fcgi?dbfrom=pubmed&id=11891286&retmode=ref&cmd=prlinks. 11891286 10.1073/pnas.052016199 PMC122593

[58] Weide T, Herrmann L, Bockau U, Niebur N, Aldag I, et al (2006) Secretion of functional human enzymes by Tetrahymena thermophila. 6: 19 Available: http://eutils.ncbi.nlm.nih.gov/entrez/eutils/elink.fcgi?dbfrom=pubmed&id=16542419&retmode=ref&cmd=prlinks. 10.1186/1472-6750-6-19 PMC1431531 16542419

[59] Turkewitz AP, Orias E, Kapler G (2002) Functional genomics: the coming of age for Tetrahymena thermophila. Trends in genetics : TIG 18: 35–40 Available: http://eutils.ncbi.nlm.nih.gov/entrez/eutils/elink.fcgi?dbfrom=pubmed&id=11750699&retmode=ref&cmd=prlinks. 11750699 10.1016/s0168-9525(01)02560-4

[60] Blackman MJ, Whittle H, Holder AA (1991) Processing of the Plasmodium falciparum major merozoite surface protein-1: identification of a 33-kilodalton secondary processing product which is shed prior to erythrocyte invasion. Molecular and biochemical parasitology 49: 35–44 Available: http://eutils.ncbi.nlm.nih.gov/entrez/eutils/elink.fcgi?dbfrom=pubmed&id=1723148&retmode=ref&cmd=prlinks. 1723148 10.1016/0166-6851(91)90128-s

[61] Kocken CHM, Withers-Martinez C, Dubbeld MA, Van Der Wel A, Hackett F, et al (2002) High-Level Expression of the Malaria Blood-Stage Vaccine Candidate Plasmodium falciparum Apical Membrane Antigen 1 and Induction of Antibodies That Inhibit Erythrocyte Invasion. Infection and immunity 70: 4471–4476 Available: http://iai.asm.org/cgi/doi/10.1128/IAI.70.8.4471-4476.2002. 12117958 10.1128/IAI.70.8.4471-4476.2002 PMC128198

[62] Gowda DC, Gupta P, Davidson EA (1997) Glycosylphosphatidylinositol anchors represent the major carbohydrate modification in proteins of intraerythrocytic stage Plasmodium falciparum. The Journal of biological chemistry 272: 6428–6439 Available: http://eutils.ncbi.nlm.nih.gov/entrez/eutils/elink.fcgi?dbfrom=pubmed&id=9045667&retmode=ref&cmd=prlinks. 9045667 10.1074/jbc.272.10.6428

[63] Gowda DC, Davidson EA (1999) Protein glycosylation in the malaria parasite. Parasitology today (Personal ed) 15: 147–152 Available: http://www.sciencedirect.com/science?_ob=ArticleURL&_udi=B6TB8-3Y8687R-8&_user=809099&_rdoc=1&_fmt=&_orig=search&_sort=d&view=c&_acct=C000043939&_version=1&_urlVersion=0&_userid=809099&md5=da54a5a92d2c9735bb2ac6e77ccc3768. 10322336 10.1016/s0169-4758(99)01412-x

[64] Garrity RR, Rimmelzwaan G, Minassian A, Tsai WP, Lin G, et al (1997) Refocusing neutralizing antibody response by targeted dampening of an immunodominant epitope. Journal of immunology (Baltimore, Md : 1950) 159: 279–289 Available: http://eutils.ncbi.nlm.nih.gov/entrez/eutils/elink.fcgi?dbfrom=pubmed&id=9200464&retmode=ref&cmd=prlinks. 9200464

[65] Tompa P (2002) Intrinsically unstructured proteins. Trends in Biochemical Sciences 27: 527–533 Available: http://eutils.ncbi.nlm.nih.gov/entrez/eutils/elink.fcgi?dbfrom=pubmed&id=12368089&retmode=ref&cmd=prlinks. 12368089 10.1016/s0968-0004(02)02169-2

[66] Tompa P (2003) Intrinsically unstructured proteins evolve by repeat expansion. BioEssays 25: 847–855 Available: http://doi.wiley.com/10.1002/bies.10324. 12938174 10.1002/bies.10324

[67] Blackman MJ (1990) A single fragment of a malaria merozoite surface protein remains on the parasite during red cell invasion and is the target of invasion- inhibiting antibodies. Journal of Experimental Medicine 172: 379–382 Available: http://www.jem.org/cgi/doi/10.1084/jem.172.1.379. 1694225 10.1084/jem.172.1.379 PMC2188181

[68] McBride JS, Heidrich HG (1987) Fragments of the polymorphic Mr 185,000 glycoprotein from the surface of isolated Plasmodium falciparum merozoites form an antigenic complex. Molecular and biochemical parasitology 23: 71–84 Available: http://eutils.ncbi.nlm.nih.gov/entrez/eutils/elink.fcgi?dbfrom=pubmed&id=2437453&retmode=ref&cmd=prlinks. 2437453 10.1016/0166-6851(87)90189-7

[69] Morgan WD, Lock MJ, Frenkiel TA, Grainger M, Holder AA (2004) Malaria parasite-inhibitory antibody epitopes on Plasmodium falciparum merozoite surface protein-1(19) mapped by TROSY NMR. Molecular and biochemical parasitology 138: 29–36 Available: http://eutils.ncbi.nlm.nih.gov/entrez/eutils/elink.fcgi?dbfrom=pubmed&id=15500913&retmode=ref&cmd=prlinks. 15500913 10.1016/j.molbiopara.2004.06.014

[70] Uthaipibull C, Aufiero B, Syed SEH, Hansen B, Patiño JAG, et al (2001) Inhibitory and blocking monoclonal antibody epitopes on merozoite surface protein 1 of the malaria parasite Plasmodium falciparum. Journal of molecular biology 307: 1381–1394 Available: http://linkinghub.elsevier.com/retrieve/pii/S0022283601945747. 11292349 10.1006/jmbi.2001.4574

[71] Burgess BR, Schuck P, Garboczi DN (2005) Dissection of merozoite surface protein 3, a representative of a family of Plasmodium falciparum surface proteins, reveals an oligomeric and highly elongated molecule. The Journal of biological chemistry 280: 37236–37245 Available: http://eutils.ncbi.nlm.nih.gov/entrez/eutils/elink.fcgi?dbfrom=pubmed&id=16135515&retmode=ref&cmd=prlinks. 16135515 10.1074/jbc.M506753200

[72] Adda CG, Murphy VJ, Sunde M, Waddington LJ, Schloegel J, et al (2009) Plasmodium falciparum merozoite surface protein 2 is unstructured and forms amyloid-like fibrils. Molecular and biochemical parasitology 166: 159–171 Available: http://eutils.ncbi.nlm.nih.gov/entrez/eutils/elink.fcgi?dbfrom=pubmed&id=19450733&retmode=ref&cmd=prlinks. 19450733 10.1016/j.molbiopara.2009.03.012 PMC2713819

[73] Smith DB, Johnson KS (1988) Single-step purification of polypeptides expressed in Escherichia coli as fusions with glutathione S-transferase. Gene 67: 31–40 Available: http://eutils.ncbi.nlm.nih.gov/entrez/eutils/elink.fcgi?dbfrom=pubmed&id=3047011&retmode=ref&cmd=prlinks. 3047011 10.1016/0378-1119(88)90005-4

[74] Khan F, Legler PM, Mease RM, Duncan EH, Bergmann-Leitner ES, et al (2012) Histidine affinity tags affect MSP1(42) structural stability and immunodominance in mice. Biotechnology journal 7: 133–147 Available: http://eutils.ncbi.nlm.nih.gov/entrez/eutils/elink.fcgi?dbfrom=pubmed&id=22076863&retmode=ref&cmd=prlinks. 22076863 10.1002/biot.201100331

